# Multilocus genotyping of *Giardia duodenalis* in Southwestern Iran. A community survey

**DOI:** 10.1371/journal.pone.0228317

**Published:** 2020-02-06

**Authors:** Abdollah Rafiei, Raheleh Baghlaninezhad, Pamela C. Köster, Begoña Bailo, Marta Hernández de Mingo, David Carmena, Esmat Panabad, Molouk Beiromvand

**Affiliations:** 1 Infectious and Tropical Diseases Research Center, Health Research Institute, Ahvaz Jundishapur University of Medical Sciences, Ahvaz, Khuzestan, Iran; 2 Department of Parasitology, School of Medicine, Ahvaz Jundishapur University of Medical Sciences, Ahvaz, Khuzestan, Iran; 3 Parasitology Reference and Research Laboratory, National Centre for Microbiology, Majadahonda, Madrid, Spain; Aga Khan University - Kenya, KENYA

## Abstract

*Giardia duodenalis* is one of the main enteric pathogens associated with diarrheal disease. In developing countries, giardiasis is a major public health concern, particularly in children under five years of age. This study aimed to evaluate the occurrence and genetic diversity of *G*. *duodenalis* causing human infections in Shushtar County, Southwestern Iran. Individual faecal specimens were collected from 1,163 individuals (male/female ratio: 0.9; age range 2–75 years) with (*n* = 258) and without (*n* = 905) gastrointestinal symptoms living in rural and urban settings during the period 2017–2018. Conventional (sucrose flotation and microscopy) methods were used for the initial detection of *G*. *duodenalis* cysts in faecal specimens. Microscopy-positive samples were confirmed by PCR amplification and sequencing of the small subunit rRNA (*ssu* rRNA) gene of the parasite. A multilocus genotyping (MLG) scheme targeting the triose phosphate isomerase (*tpi*), the glutamate dehydrogenase (*gdh*), and the beta-giardin (*bg*) genes was used for genotyping purposes. *Giardia duodenalis* cysts were detected in 7.7% (90/1,163) of samples by microscopy, of which 82 were confirmed by *ssu*-PCR. Successful amplification and sequencing results were obtained for 23.2% (19/82), 9.8% (8/82), and 8.5% (7/82) of the confirmed samples at the *tpi*, *gdh*, and *bg* loci, respectively. MLG data for the three loci were available for two samples only. Out of the 24 samples genotyped at any loci, 50% (12/24) were identified as assemblage A and the remaining half as assemblage B. Overall, AII was the most prevalent sub-assemblage detected (41.7%, 10/24), followed by BIII (25.0%, 6/24), discordant BIII/BIV (5/24) or AII/AIII (2/24) sequences, and BIV (1/24). No significant correlation was demonstrated between a given assemblage/sub-assemblage and the occurrence of clinical symptoms. No genotypes adapted to animal hosts other than humans (e.g. assemblages C-F) were found circulating in the investigated human population, suggesting that transmission of human giardiasis in this Iranian region is primarily of anthroponotic nature. Further molecular-based studies are needed to confirm and expand these results, and to ascertain the presence and public health relevance of the parasite in environmental (e.g. drinking water) samples.

## Introduction

Parasitic diseases are public health problems in poor-resourced communities living in low and middle-income countries (LMICs) [1]. Giardiasis and cryptosporidiosis, the two most important waterborne diseases of protozoan origin, are a frequent cause of diarrheal outbreaks throughout the world [2–5]. Because giardiasis has a negative impact on child health, pregnancy and worker productivity and affect their socio-economic development, the disease was included in the WHO Neglected Diseases Initiative in 2004. *Giardia duodenalis* is a protozoan that colonize/infect the small intestine of humans and other vertebrates [6]. It is estimated that near 200 million people in Africa, Asia, and Latin America have symptomatic giardiasis every year [7], although the number of subclinical infections is much higher. The parasite has a direct life cycle comprising two developmental stages, the trophozoite and the cyst. The trophozoite is the replicative stage and causes the clinical symptoms of giardiasis, whereas the cyst is the infective stage and are shed in the faeces of infected individuals [8, 9]. *Giardia duodenalis* infection occurs by accidental ingestion of infectious cysts through contaminated food or water, or by direct contact with infected humans or animals [8].

Clinical giardiasis is particularly prevalent among children under five years of age, the elderly and immunocompromised patients [10, 11]. The prevalence of the infection has been reported at 2–3% in industrialized countries and at 20–30% in LMICs [12]. Variations in the occurrence rate of giardiasis have been attributed to different factors such as the sensitivity of the diagnostic tool used, the geographical location, and the socio-demographic features of the studied population [13]. Of the eight *G*. *duodenalis* assemblages (A to H) currently recognized (which are all morphologically similar and difficult to differentiate), A and B are the main assemblages causing infections in humans as well as other mammal species [14, 15]. Multilocus genotyping (MLG) approaches, based on the amplification and sequencing of the glutamate dehydrogenase (*gdh*), the triose phosphate isomerase (*tpi*), and the beta-giardin (*bg*) genes of the parasite represent a powerful tool to systematically ascertain the genetic diversity within *G*. *duodenalis*. Accordingly, assemblage A has been classified into three distinct (AI-AIII) sub-assemblages [16, 17], whereas assemblage B has been subdivided into two (BIII-BIV) sub-assemblages [8]. The most common sub-assemblages in humans are AII, BIII, and BIV, although their frequencies differ largely depending on the geographical region and sub-population considered [16]. Many studies have been conducted to clarify the potential correlation between *Giardia* assemblages and clinical symptoms; however no clear associations have been demonstrated yet [6].

In Iran, giardiasis is one of the main parasitic diseases in humans [18]. The prevalence of *G*. *duodenalis* was estimated at 1.2% to 38.0% in Iranian populations [1]. Molecular studies carried out to date, although limited, indicate that assemblage A is more prevalent than assemblage B both in symptomatic and asymptomatic populations [1, 19–21]. However, in these studies assemblage identification was based either on restriction fragment length polymorphism (PCR-RFLP) analysis or PCR and sequencing of a single genetic locus. To the best of our knowledge, only one study by Razmjou et al., presented at the 27^th^ European Congress of Clinical Microbiology and Infectious Diseases, attempted to identify *G- duodenalis* genotypes of human origin by MLG in the country [22]. Therefore, this study was conducted to assess the occurrence and molecular diversity of *G*. *duodenalis* in rural and urban populations in Shushtar County, southwestern Iran, using an MLG approach. No such studies have been conducted in this region of the country to date.

## Materials and methods

### Ethics approval and consent to participate

The study protocol was reviewed and approved by the Ethics Committee of Ahvaz Jundishapur University of Medical Sciences (Approval No IR.AJUMS.REC.1397.461). The study aims and the procedures involved were explained to, and written informed consent was obtained from voluntary participants.

### Study area

Shushtar County (32° 02’ N 48° 51’ E) is one of the 27 counties conforming the Khuzestan Province in Southwestern Iran. It is approximately 150 meters above sea level and extends over an area of 2,436 km^2^. Based on the 2016 Iranian census, reported population is about 192,028 inhabitants distributed into two urban (Markazi and Shoaybiyeh) and 6 rural (Sardarabad, Western Shoaybiyeh, Eastern Shoaybiyeh, Shahid Modares, Northern Mianab, and Southern Mianab) districts. Most of the rural inhabitant usually keep domestic animals including dogs, cattle, goats, and chickens, at homes. Shushtar County has a Mediterranean climate, with warm and dry summers and mild winters.

### Study population

A prospective, community-based epidemiological study was conducted by simple random sampling among rural and urban people in Shushtar County during 2017–2018. Informative meetings were held with local education authorities and asked for collaboration. Voluntary participants were provided with polystyrene plastic flask and requested to provide individual stool samples. A standardized questionnaire covering basic sociodemographic (age, gender, residency, and household size) and clinical (occurrence of gastrointestinal symptoms) data was completed by researchers interviewing participating individuals at the moment of sampling. Collected stool samples were transported in refrigerated containers or insulated boxes to the Department of Parasitology, School of Medicine, Ahvaz Jundishapur University of Medical Sciences (Ahvaz) for further microbiological and molecular analyses.

### Sample processing, detection and isolation of *G*. *duodenalis* cysts

Direct microscopy with formalin-ether concentration technique was used as screening test in all the stool samples collected. In those samples with a *G*. *duodenalis*-positive result after microscopy examination the sucrose-flotation method was performed to isolate the cysts of the parasite as described previously with slight modifications on centrifugation speed and volume of sucrose used [23]. Briefly, approximately 5 g of faecal material was emulsified in 50 mL of distilled water, filtered through a four-layer gauze, and centrifuged at 1000 ×g for five min. After removing the supernatant, the remaining sediment was re-suspended in 30 mL of distilled water, mixed thoroughly for one min, and added to the top of a 15 mL cold sucrose solution (1 M). After centrifugation at 800 ×g for 5 min, the top two layers containing the *G*. *duodenalis* cysts were transferred into a clean Falcon tube and the volume adjusted to 50 mL with distilled water. After three consecutive centrifugations at 1000 ×g for five min, an aliquot of the obtained pellet was examined under the light microscope at ×400 magnification. The remaining sediment was kept at −20 °C for downstream molecular analyses.

### DNA extraction

Faecal sediments were subjected to seven freeze-thaw cycles consisting of 7 min in liquid nitrogen and 7 min in a boiling water bath to facilitate cyst rupture and parasite DNA extraction. Genomic DNA was purified using the QIAamp DNA stool mini kit (Qiagen, Hilden, Germany) according to the manufacture’s protocol. DNA was eluted in 200 μL of AE buffer and stored at −20 °C until use.

### Molecular detection and characterization of *Giardia duodenalis*

Faecal samples with a positive result for *G*. *duodenalis* at microscopy were re-assessed by molecular (including PCR and sequencing) methods. Confirmation of the presence of the parasite was conducted by a nested PCR protocol to amplify a ~130-bp fragment of the small subunit of ribosomal RNA (*ssu-*rRNA) gene of the parasite as described previously [24]. Primary and secondary reactions were carried out using the outer primer pair RH11 (5´–CATCCGGTCGATCCTGCC–3´) and RH4 (5´–AGTCGAACCCTGATTCTCCGCCAGG–3´), and the inner primer set GiarF (5´–GACGCTCTCCCCAAGGAC–3´) and GiarR (5´–CTGCGTCACGCTGCTCG–3´), respectively. Reaction mixtures included of 2 μL of template DNA both in the primary and secondary PCR reactions. Cycling conditions were identical for the first and second round of PCR, and consisted of an initial denaturation at 95 °C for 3 min, followed by 35 cycles of amplification (denaturation at 95 °C for 30 s, annealing at 55 °C for 30 s, and elongation at 72 °C for 60 s), and a final extension at 72 °C for 7 min.

DNA samples with a positive result at *ssu*-PCR were subsequently investigated using a multilocus genotyping (MLG) scheme based on the amplification of partial sequences of the *gdh*, the *bg*, and the *tpi* genes of *G*. *duodenalis*. To amplify a ~432-bp fragment of the *gdh* gene, a semi-nested PCR was performed as described elsewhere [25]. The protocol included the outer primer set GDHeF (5´–TCAACGTYAAYCGYGGYTTCCGT–3´) and GDHiR (5´–GTTRTCCTTGCACATCTCC–3´), and the inner primer set GDHiF (5´–CAGTACAACTCYGCTCTCGG–3´) and GDHiR (5´–GTTRTCCTTGCACATCTCC–3´). Both PCR reaction mixtures included 5 μl of template DNA. Both the primary and secondary PCRs were carried out as follows: an initial denaturation step of 95 °C for 3 min, followed by 35 cycles of 95 °C for 30 s, 55 °C for 30 s, and 72 °C for 60 s, with a final extension of 72 °C for 7 min.

For the *bg* gene, a partial sequence of ~511 bp was amplified using the outer primer pair G7 (5´–AAGCCCGACGACCTCACCCGCAGTGC–3´) and G759 (5´–GAGGCCGCCCTGGATCTTCGAGACGAC–3´) and the inner primer pair BG-F (5´–GAACGAACGAGATCGAGGTCCG–3´) and BG-R (5´–CTCGACGAGCTTCGTGTT–3´) as described previously [26]. Reaction mixtures included of 3 μL of template DNA in both PCR rounds. The primary PCR was performed as follows: initial heat activation step at 96 °C for 5 min, 40 cycles of 95 °C for 30 s, 50 °C for 30 s, and 72 °C for 60 s, with a final extension of 72 °C for 7 min. Cycling conditions for the secondary PCR included an initial heat activation step at 96 °C for 5 min followed by 35 cycles of 96 °C for 45 s, 55 °C for 30 s, 72 °C for 45 s, with a final extension of 72 °C for 7 min.

For the *tpi* gene, a ~530-bp fragment was amplified using the outer primer pair AL3543 (5´–AAATIATGCCTGCTCGTCG–3´) and AL3546 (5´–CAAACCTTITCCGCAAACC–3´), and the inner primer pair AL3544 (5´–CCCTTCATCGGIGGTAACTT–3´) and AL3545 (5´–GTGGCCACCACICCCGTGCC–3´) as described by Sulaiman et al. (2003) [27]. The primary PCR conditions consisted of an initial denaturation step at 94 °C for 5 min followed by 35 cycles of 94 °C for 45 s, 50 °C for 45 s, and 72 °C for 60 s, with a final extension of 72 °C for 10 min. The conditions for the secondary PCR were identical to the primary PCR.

All PCR reactions described above were carried out in a FlexCycler^2^ PCR Thermal Cycler (Analytik Jena, Jena, Germany). PCR mixtures (25 μL) contained 12.5 μL of 2× Master Mix RED already including Taq DNA polymerase (Ampliqon-Biomol, Hamburg, Germany), 200 nM of each forward and reverse primer, and nuclease-free water. Negative (no DNA template) and positive (known *G*. *duodenalis* PCR-positive sample) controls were routinely included in all PCR runs. Secondary PCR products were analysed by electrophoresis on 1.5% agarose gels (Sinacolon, Tehran, Iran) and visualized after ethidium bromide staining.

### DNA sequencing and phylogenetic analyses

Obtained *gdh*, *bg*, and *tpi* amplicons of the right size were directly sent for sequencing in both directions using the corresponding internal primer pairs at Microsynth AG (Balgach, Switzerland). Raw sequences were visually inspected using the free software Chromas version 2.1 as quality control and to detect the presence of single nucleotide polymorphisms (SNPs) including double peaks. Obtained consensus sequences were compared with those previously deposited at the National Centre for Biotechnology Information (NCBI) using the BLAST tool (http://www.ncbi.nlm.nih.gov/blast). Assignment of assemblages and sub-assemblages was carried out by sequence alignment using ClustalW in MEGA 6.0 (www.megasoftware.net). A phylogenetic analysis was performed on the sequences obtained in the present survey at the *gdh*, *bg*, and *tpi* loci and previously published sequences of human and animal origin retrieved from GenBank using the Neighbor-Joining (NJ) method. Sequences including heterozygous (di-nucleotide) sites were excluded from the analyses in order to avoid distorting the topology of the phylogenetic trees. Evolutionary relationships were calculated by the Kimura-2-parameter model in MEGA 6.0. The reliability of these trees was assessed by using the bootstrap method with 1,000 pseudoreplicates; only values >70% were reported. Representative sequences at the *gdh*, *bg*, and *tpi* loci generated in the present survey were deposited in GenBank under accession numbers LC504257 to LC504276 and LC505038 to LC505051.

### Statistical analyses

Data analysis was performed using the SPSS 22 software (SPSS Inc., Chicago, IL, USA). The Chi-squared test was used to compare the participant’s socio-demographic parameters and analyse statistical differences between the infected and non-infected individuals.

## Results

In the present epidemiological study, individual faecal samples were collected from a total of 1,163 volunteers during the sampling period. The male/female ratio was 0.9. The age range of the participating individuals was 2–75 years (mean: 27.3; SD: 19.3). Regarding the place of residence, 43.5% (506/1,163) of the recruited subjects lived in rural settings. Individuals in the age groups <10 years and 31 to 40 years were the most represented in the surveyed population. Most participants lived in households with 4 to 6 (66.0%, 767/1,163) or above (11.3%, 131/1,163) members, respectively. Gastrointestinal symptoms including diarrhoea, bloating and abdominal pain were reported by 22.2% (258/1,163) of the participants at the moment of sampling. Symptoms were predominantly seen (7.3%, 85/1,163) in individuals younger than 10 years of age.

Using conventional microscopy methods, the overall prevalence of *G*. *duodenalis* infection was estimated at 7.7% (90/1,163, 95% confidence interval: 6.2% to 9.3%). The distribution of the infection according to the variables (gender, age, area of residence, household size, symptomatology) considered in the study are summarized in Table 1. Briefly, males (9.7%) were significantly more infected by *G*. *duodenalis* than females (5.9%) (*p* = 0.014). *Giardia* infections were more likely to occur in children under 10 years of age (*p* < 0.001). No significant correlation was found between the occurrence of giardiasis and the number of family members in each household. Human cases of giardiasis were more frequently found in rural (11.7%, 59/506) than in urban (4.7%, 31/657) areas (*p* < 0.001). Approximately three out of four cases of giardiasis (74/90) occurred in households with four or more persons. *Giardia duodenalis* infections were mostly subclinical (57.8%, 52/90) (*p* < 0.001). Diarrhoea was reported in 10.0% (9/90) of the *Giardia*-infected individuals.

**Table 1:** Main sociodemographic and clinical characteristics of the surveyed human population. Shushtar County (Iran), 2017–2018. Estimated p-values are indicated. Regarding the age, the infection rate was compared within the age groups.

		Non infected (*n* = 1,073)		Infected^a^ with *G*. *duodenalis* (*n* = 90)		*P*
Category	Group	No.	%	No.	%	
Gender	Male	500	90.3	54	9.7	0.014
	Female	573	94.1	36	5.9	
Age group (years)	<10	270	88.5	35	11.5	<0.001
	10–20	162	86.6	25	13.4	
	21–30	151	93.8	10	6.2	
	31–40	195	95.6	9	4.4	
	41–50	149	95.5	7	4.5	
	>50	146	97.3	4	2.7	
Area of residence	Rural	447	88.3	59	11.7	<0.001
	Urban	626	95.3	31	4.7	
Family size (no. of members)	<4	249	94.0	16	6.0	0.165
	4–6	708	92.3	59	7.7	
	≥7	116	88.5	15	11.5	
Clinical symptoms	Asymptomatic	1,071	93.0	81	7.0	<0.001
	Diarrhea	2	18.2	9	81.8	

^a^ As diagnosed by conventional microscopy methods.

http://dx.doi.org/10.17504/protocols.io.bapfidjn

### Molecular detection and genotyping data

Of the 90 *Giardia*-positive cases detected by microscopy examination, 82 (91.1%) were confirmed using *ssu*-PCR. Under the MLG scheme adopted in the present study, successful PCR amplification and sequencing data were achieved for 9.8% (8/82), 8.5% (7/82) and 23.2% (19/82) of the confirmed samples tested at the *gdh*, *bg*, and *tpi* loci, respectively. A total of 24 *Giardia*-positive samples were genotyped at least at a single locus. MLG data at the three loci tested were available for two samples only (Table 2). Sequencing results revealed that assemblages A and B were equally present (50%, 12/24 each) in the surveyed human population. No A+B mixed infections, nor host-specific assemblages of canine, feline, or livestock (C-F) origin were detected. Overall, AII was the most prevalent sub-assemblage detected (41.7%, 10/24), followed by BIII (25.0%, 6/24), discordant BIII/BIV (5/24) or AII/AIII (2/24) sequences, and BIV (1/24).

**Table 2:** Multilocus genotyping results of the 24 *Giardia*-positive samples successfully genotyped at least at one of the three loci investigated. Shushtar County (Iran), 2017–2018.

Isolate ID	*gdh*	*bg*	*tpi*	Assigned genotype
**10**	BIII/BIV	–	–	BIII/BIV
**15**	AII	AIII	AII	AII/AIII
**16**	AII	–	AII	AII
**18**	–	–	BIII	BIII
**19**	AII	–	–	AII
**20**	BIII/BIV	B	BIII	BIII/BIV
**22**	–	–	AII	AII
**33**	–	B	BIII	BIII
**38**	–	B	BIII	BIII
**41**	–	B	BIII/BIV	BIII/BIV
**42**	–	–	AII	AII
**47**	AII	–	–	AII
**48**	BIII/BIV	–	BIII	BIII/BIV
**54**	AII	–	–	AII
**61**	–	–	BIII	BIII
**62**	–	–	AII	AII
**67**	–	AII	–	AII
**68**	–	–	AII	AII
**69**	–	–	BIII	BIII
**71**	–	AIII	AII	AII/AIII
**77**	–	–	BIV	BIV
**80**	–	–	BIII/BIV	BIII/BIV
**81**	–	–	BIII	BIII
**82**	–	–	AII	AII

http://dx.doi.org/10.17504/protocols.io.bapgidjw

Out of the eight *gdh* sequences identified five were assigned to the sub-assemblage AII and three were discordant BIII/BIV results. Of the five AII sequences, one showed 100% identity with a previously described reference sequence (GenBank accession number L40510) and the remaining four varied from one to two SNPs with L40510. The three BIII/BIV sequences had 4–14 SNPs, some of them corresponding to polymorphic (double peaks) positions (Table 3).

**Table 3:** Diversity, frequency, and main molecular features of *Giardia duodenalis* sequences at the *gdh*, *bg*, and *tpi* loci generated in the present study. GenBank accession numbers are provided. Shushtar County (Iran), 2017–2018.

Locus	Assemblage	Sub-assemblage	No. of isolates	Reference sequence	Stretch	Single nucleotide polymorphisms	GenBank accession number
***gdh***	A	AII	2	L40510	67–496	C492T	LC504257
			1	L40510	67–496	C85Y, C492T	LC505038
			1	L40510	67–496	T469W, T492Y	LC505039
			1	L40510	67–496	None	LC504259
	B	BIII/BIV	1	L40508	79–491	T186C, C276T, T390C, A441G	LC504260
			1	L40508	79–491	T138C, C231G, C258T, C276T, A462G, T496Y	LC504261
			1	L40508	55–491	C63T, C75T, C123Y, T135Y, T183C, G186R, C255Y, C273Y, C345Y, T366Y, C372Y, T387C, C396Y, A438R	LC504262
***bg***	A	AII	1	AY072723	63–500	None	LC504266
		AIII	2	AY072724	63–500	None	LC504267
	B	B	1	AY072727	63–500	A183G	LC504265
			1	AY072727	63–526	C123T	LC504263
			1	AY072727	63–526	A119R, C165T, C249T, G289A, C309T, C518T	LC504264
			1	AY072727	105–550	A183G, C256Y, C348T, C564Y	LC505040
***tpi***	A	AII	2	U57897	288–798	None	LC504269
			2	U57897	304–790	C374M	LC505041
			1	U57897	290–790	C723T	LC504270
			1	U57897	275–805	C287G, G705A	LC504271
			1	U57897	294–807	G726A	LC504272
			1	U57897	300–790	T602Y, C635Y, G746A	LC505051
	B	BIII	1	AF069561	318–795	T294C, G370A	LC504274
			1	AF069561	318–775	G105A	LC504273
			1	AF069561	322–775	C30Y, C104T, C107Y, C137Y, C179Y, C210Y, T377Y	LC505042
			1	AF069561	318–775	A135R, G305R, C336Y	LC505043
			1	AF069561	327–775	T264C	LC504275
			1	AF069561	330–775	T264Y, T294Y	LC505045
			1	AF069561	318–775	C34T, T69Y, G105A	LC505046
			1	AF069561	327–775	C108T	LC504276
		BIV	1	AF069560	297–775	C63M, A174G, A393G	LC505049
		BIII/BIV	1	AF069561	295–775	A41R, C46Y, T57Y, T131Y, T134Y, A162R, C164Y, C171Y, A176G, C298Y, A395G	LC505044
			1	AF069561	295–775	A5R, T57Y, A60R, G128R, T131Y, T134Y, A176G, C194Y, G200R, A395G, G422R	LC505050

A total of seven *bg* sequences were generated, of which one was identified as AII (showing 100% identity with AY072723) and two as AIII (showing 100% identity with AY072724). The four sequences assigned to assemblage B at this locus differed by one to six SNPs from reference sequence AY072727. Ambiguous positions were identified in two of these B sequences, but at a mch lower rate than those found at the *gdh* locus (Table 3).

Multiple sequence alignment analyses of the 19 *tpi* sequences available revealed the presence of sub-assemblages AII in eight of them, of which one showed 100% identity with reference sequence U57897and the remaining seven differed from it by 1–3 SNPs. Ambiguous (double peaks) positions were detected in two sequences (Table 3). Similar to what was observed at the *gdh* locus, a high genetic diversity was also present within the *tpi* sequences assigned to assemblage B. All BIII sequences differed among them and from reference sequence AF069561 by 1–7 SNPs. The only BIV sequence identified at this locus varied from reference sequence AF069560 by 3 SNPs, whereas two additional sequences showed discordant genotype BIII/BIV results, very likely representing mixed infections by both sub-assemblages of the parasite.

**Fig 1:**
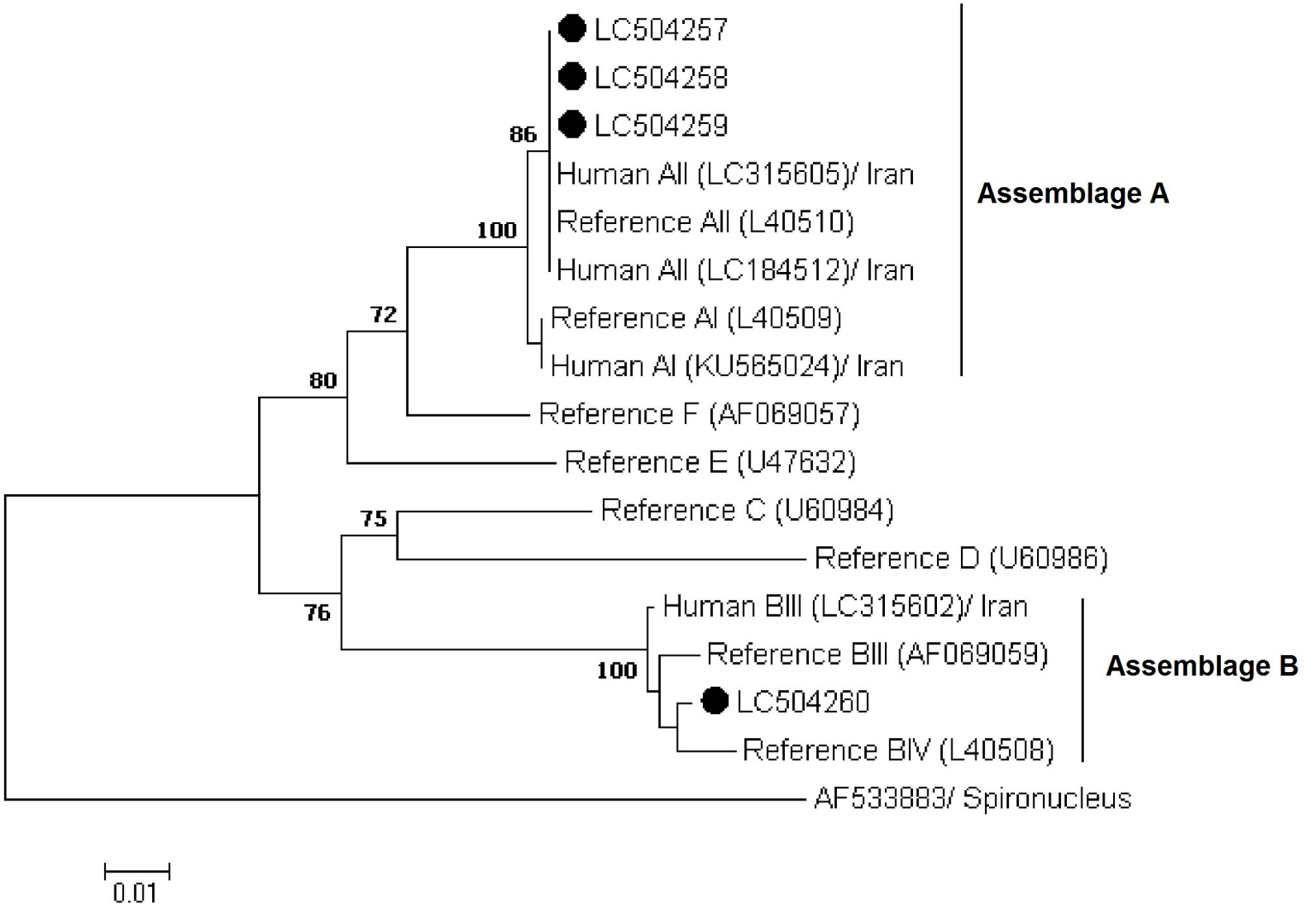
Phylogenetic relationships of *Giardia duodenalis* isolates inferred by the neighbor-joining analysis of the glutamate dehydrogenase (*gdh*) nucleotide sequences. Filled black circles represent *gdh* sequences generated in the present study. Previously published sequences of human origin and reference sequences retrieved from GenBank were included in the analyses for comparative purposes. *Spironucleus vortens* was used as outgroup taxa.

**Fig 2:**
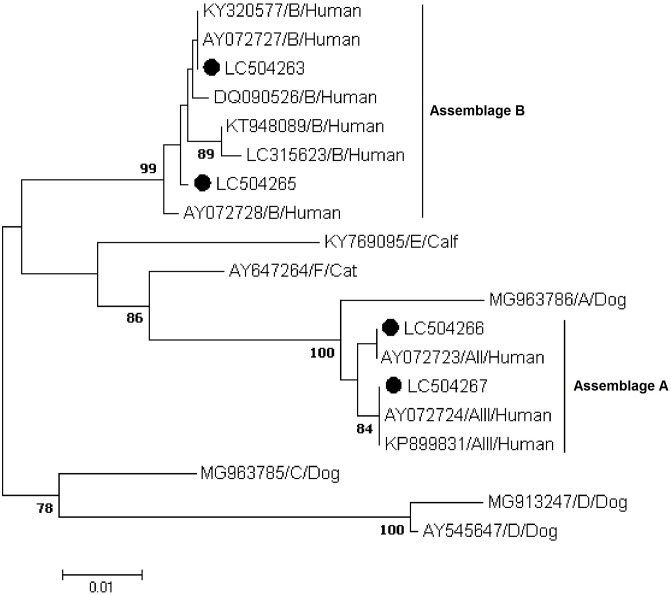
Phylogenetic relationships of *Giardia duodenalis* isolates inferred by the neighbor-joining analysis of the beta-giardin (*bg*) nucleotide sequences. Filled black circles represent *gdh* sequences generated in the present study. Previously published sequences of human origin and reference sequences retrieved from GenBank were included in the analyses for comparative purposes.

**Fig 3:**
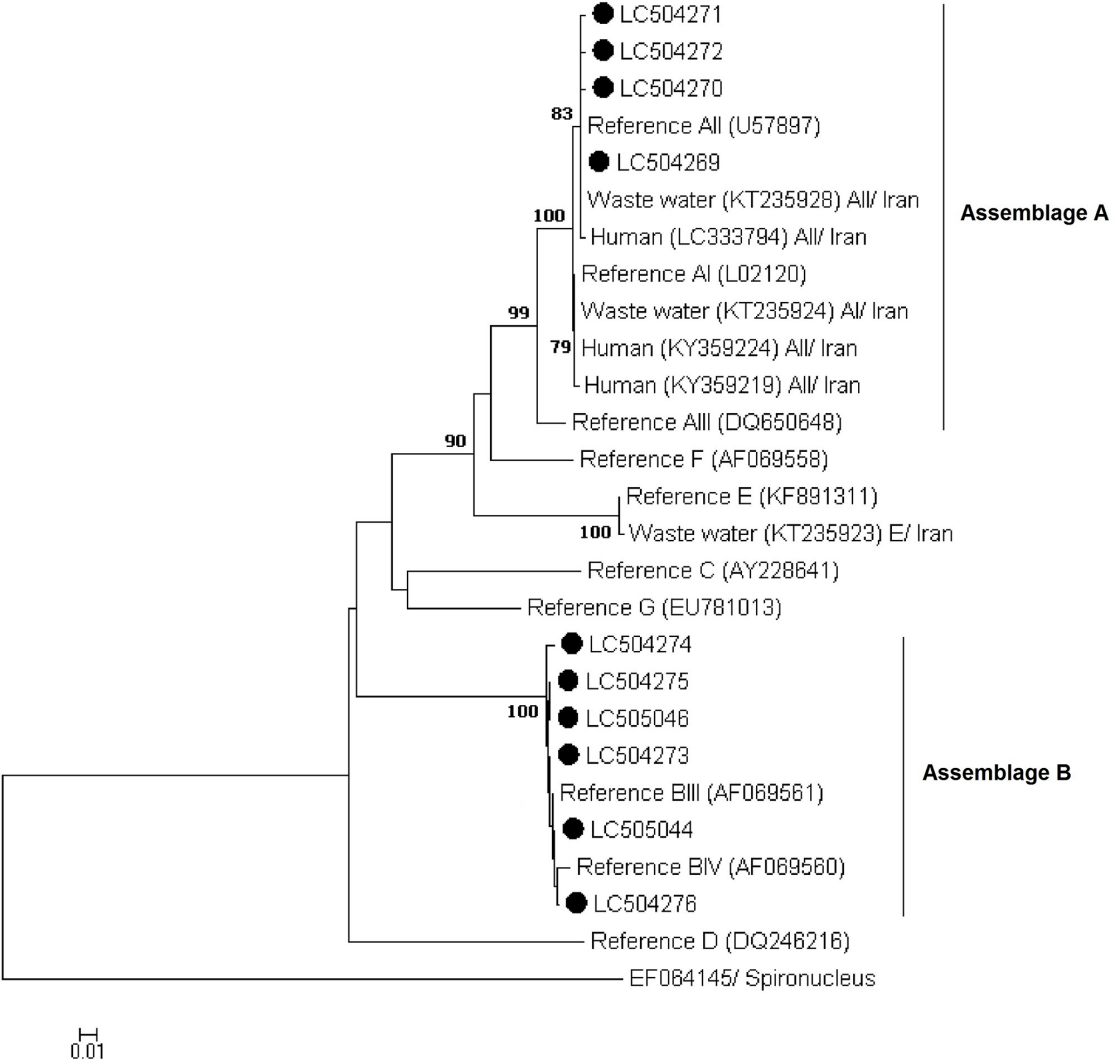
Phylogenetic relationships of *Giardia duodenalis* isolates inferred by the neighbor-joining analysis of the triose phosphate isomerase (*tpi*) nucleotide sequences. Filled black circles represent *tpi* sequences generated in the present study. Previously published sequences of human origin and reference sequences retrieved from GenBank were included in the analyses for comparative purposes. *Spironucleus vortens* was used as outgroup taxa.

Phylogenetic analyses using the NJ method revealed that all sequences generated in the present study at the *gdh*, *bg*, and *tpi* loci grouped in well-defined clusters with appropriate reference sequences and previously published sequences retrieved from GenBank (Figs 1 to 3).

## Discussion

Giardiasis is one of the main causes of diarrhoea, particularly in children under five years of age in the developing world [28]. In Iran, human giardiasis has been reported at infection rates ranging from 5% to 23% in different parts of the country [29]. Documented animal prevalences were 5–16% in goats, 6–20% in sheep, and 4% in cattle [30, 31]; however, molecular epidemiological surveys based on MLG schemes are lacking in the country. The available molecular data on giardiasis in Iran are limited to few studies conducted by PCR-RFLP and nested PCR at the *gdh* or *tpi* locus [1, 21, 32, 33]. These surveys coincide to identify assemblage A, sub-assemblage AII as the predominant *G*. *duodenalis* genetic variant circulating in Iranian human population. Khuzestan Province, with a prevalence ranging from 2% to 11%, is considered an endemic area for giardiasis in Iran [19, 20, 32, 34–36]. However, available epidemiological data are restricted to people living in urban settings, with those inhabiting rural areas being largely neglected. In addition, previous studies have relied on microscopy examination only, and those using molecular approached were based in single-locus analyses. Therefore, little is known about the frequency and genotypic diversity of *G*. *duodenalis* in human and animal populations in the province [19, 20, 34].

An overall *G*. *duodenalis* prevalence of near 8% was estimated by microscopy examination in the surveyed general population of Khuzestan Province. This figure is well in the range with those (2–11%) previously reported in this geographical area [19, 20, 32, 34, 35]. In this regard, an average prevalence of 30% has been estimated in a recent systematic review and meta-analysis study focusing in Iranian children populations [37]. Of note, eight out of the 90 *Giardia*-positive cases detected by microscopy examination could not be confirmed by *ssu*-PCR. This discrepancy may be due to a number of factors including insufficient amount or suboptimal quality of parasite DNA, or inefficient removal of PCR inhibitors during the DNA extraction and purification process. It is important to bear in mind that the *G*. *duodenalis* infection rate reported here is very likely a conservative estimation of the true prevalence of the disease, as the diagnostic sensitivity of conventional microscopy is limited. Based on these data, giardiasis was more frequently detected in children under 10 years of age living in rural settings (28.9%; 26/90). Poorer sanitation facilities and hygienic conditions, limited access to drinking water and more crowded living conditions may account for this higher prevalence of the disease in rural areas compared to those detected in urban areas. In a few studies, *G*. *duodenalis* cysts have been identified in surface waters intended for human consumption in north and southwestern Iran [38, 39].

This study presents novel data on the diversity of *G*. *duodenalis* among both rural and urban populations in Khuzestan Province, southwestern Iran. Success amplification rates at the *gdh*, *bg*, and *tpi* loci varied from 8% to 23%. These figures were lower than those (51%- 58%) achieved by our research team in a previous survey conducted in Andimeshk County, north of Khuzestan Province [34]. The reasons for these discrepancies are unclear, but limited sensitivities of PCRs based in single-copy genes (as it is the case for the *gdh*, *bg*, and *tpi* loci used in our MLG scheme) may explain, at least partially, this situation. This is probably the main limitation of the present study. It should be noted that previous molecular studies based on ribosomal RNA gene sequence analysis were able to successfully differentiate *G*. *duodenalis* sub-assemblages AI and AII from assemblage B [40]. In this regard, in their study Weiss et al. amplified a183-bp DNA fragment of the *ssu* rRNA gene, a sequence 40% longer than the fragment (130-bp) amplified in our *ssu*-PCR protocol. In practical terms this means that amplicons obtained by Weiss et al. were more informative (in terms of genetic diversity) than those obtained in the present study. Despite this drawback, single-copy genes provide the necessary discriminatory power to differentiate among different genetic variants within *G*. *duodenalis* [41]. This is supported by the fact that the *ssu*-PCR (based on a multi-copy gene) used for confirmatory purposes in the present survey was able to amplify a much higher number (*n* = 82) of *Giardia*-positive samples. The very same problem has been reported in similar studies targeting schoolchildren populations in endemic areas in Angola and Ethiopia [42, 43].

In the present study *G*. *duodenalis* assemblages A and B were found at identical proportions in the investigated population. The reported geographical distribution and frequency of *G*. *duodenalis* assemblages/sub-assemblages in Iran varies according to the region of the country considered. Thus, assemblage A has been more prevalently found in the north [21], south [18, 44], west [45], southwest [19], and centre of Iran [1], while some studies carried out in southwestern [46] and north western [47, 48] regions of the country reported assemblage B as the predominant assemblage. These discrepancies may be associated to differences in the total number of samples successfully genotyped in each region, or to specific socio-economic and epidemiological factors of the population studied. In many rural areas of Shushtar County, people keep animals at homes [36], suggesting that companion animals may play a role as source of human infections. Previous studies have shown that assemblage A can be divided into sub-assemblages of AI-AIII, and assemblage B into sub-assemblages BIII and BIV [49]. Sub-assemblages AI, AII, BIII, and BIV have all been reported causing infections in humans and other animal host species, and are therefore considered zoonotic [15]. However, the fact that no host-adapted canine (C, D), livestock (E) or feline (F) genotypes have been identified in the present study seem to suggest that most of the *Giardia* infections reported here are of anthroponotic origin. This hypothesis should be confirmed in future molecular studies investigating the occurrence and molecular diversity of *G*. *duodenalis* infections in production and companion animals in this region.

Association between *Giardia* assemblages and clinical symptoms has been investigated in several molecular surveys with discrepant results [34, 50–53]. Host adaptive immunity driven by *G*. *duodenalis*-assemblage specific responses may play a role in the observed differences. In the current study, no significant association was observed between a given assemblage of the parasite and the occurrence of clinical manifestations. Of the 10 cases infected with sub-assemblages AII, one case had diarrhea. This gastrointestinal condition was also present in one out of the six cases infected with sub-assemblage BIII. In an earlier molecular epidemiological study based on *gdh*-PCR-RFLP conducted in the Khuzestan Province mixed assemblage A+B infections were allegedly reported in 54.0% of the investigated isolates [46]. In contrast, we did not find mixed infections involving different assemblages of the parasite by PCR and sequencing methods. These large differences are likely due to the inherent difficulty of interpreting band profiles after restriction enzyme digestion. Analysis of the sequences obtained at the *gdh* locus allowed the identification of sub-assemblage AII and discordant genotype BIII/BIV results in 5 (62.5%) and 3 (37.5%) isolates, respectively. Azcona-Gutierrez et al. (2017) genotyped 90 human isolates of *G*. *duodenalis* at the *gdh* and *bg* genes, reporting discordant genotyping results in one (6%) isolate [54]. In another study conducted by Skhal et al. (2017) discordant genotype results BIII/BIV were reported in four (10%) isolates [55]. Despite the fact that *Giardia* is believed to be a strictly clonal (asexual) organism, there is evidence for recombination at the telomeres (but not the internal regions) of some of the chromosomes of the parasite [9, 56]. Additional evidence of genetic recombination has been demonstrated at the cellular level in cultured assemblage B trophozoites and in cysts from clinical samples [57], and at the population level within sub-assemblages BIII and BIV [58]. Taken together, these findings may explain the occurrence of discordant genotypes BIII/BIV, as those described in the present survey.

The predominance of sub-assemblage AII in this study confirms previous genotyping data reported from the same region [19, 32, 34], but it is inconsistent with those found by Rafiei et al. showing that sub-assemblage BIII was the predominant genotype among symptomatic and asymptomatic patients [46]. This discrepancy may be due to differences in the transmission route of the parasite, the studied regions, and the studied population including their life style and personal hygienic habits. Sequencing at the *bg* locus revealed sub-assemblages AII and AIII, and assemblage B in 1 (14.3%), 2 (28.6%), and 4 (57.1%) isolates, respectively. The AII and two of the three AIII isolates showed 100% homology with their specific reference sequences, while the four assemblage B sequences presented a much higher degree of genetic diversity among them. Previous assemblage A sequence analyses have demonstrated approximately 1% genetic divergence between AI and AII sequences, which represent ∼100,000 SNPs [56]. Assemblage A shows large differences in allelic sequence heterozygosity (ASH). Maximum values of 0.25% to 0.35% correspond to sub-assemblage AII isolates AS-98 and AS-175, whereas and ASH <0.01% has been reported for assemblage AI isolate WB. Reasons for this 100-fold difference in ASH values between sub-assemblages are still unknown [6].

In the current study, 65.6% of the surveyed population lived in rural settings. However, the fact that assemblage B and sub-assemblage AII, but not animal-specific sub-assemblage AI, were found circulating in the general population investigated in the present study strongly suggests that giardiasis in the Shushtar County is acquired directly or indirectly from human sources.

## Conclusions

This is the first published study conducted by MLG genetically characterize *G*. *duodenalis* human isolates in Khuzestan Province, Iran. Results of our MLG analysis indicated a similar frequency of assemblages A and B in the human community living in Khuzestan Province. The results demonstrated no correlation between clinical symptoms and the *G*. *duodenalis* assemblage involved in the infection. Lack of sub-assemblage AI in the surveyed population support the hypothesis that most human infections in this geographical area are acquired though anthroponotic sources. Further molecular-based epidemiological studies are needed to corroborate the extent of the findings reported here.

## Abbreviations

°C: Centigrade
*bg*: beta-giardin
BLAST: *Basic Local Alignment Search Tool*
bp: Base pair
DNA: Deoxyribonucleic acid
g: Gram
*gdh*: Glutamate dehydrogenase
km^2^: Square kilometre
LMICs: living in low and middle-income countries
M: Molar
MEGA: Molecular Evolutionary Genetics Analysis
min: Minute
mL: Millilitre
MLG: Multilocus genotyping
NCBI: National Centre for Biotechnology Information
NJ: Neighbor-Joining
*p*: probability
PCR-RFLP: Polymerase chain reaction- restriction fragment length polymorphism
S: Second
SD: Standard deviation
SNP: Single-nucleotide polymorphism
SPSS: Statistical Package for the Social Sciences
*ssu-*rRNA: small subunit of ribosomal RNA
*tpi*: Triose phosphate isomerase
WHO: World Health Organization
μL: *Microliter*
